# On Diseases Erroneously Considered, and Treated as Syphilitic

**Published:** 1815-10

**Authors:** Richard Carmichael

**Affiliations:** Dublin


THE LONDON
### Medical and Physical Journal.

4 OF VOL. XXXIV.] OCTOBER, 1815. [NO. 200.

"For many fortunate discoveries in medicine, and for the detection of numerous errors, the world is indebted to the rapid circulation of Monthly Journals; and there never existed any work to which the faculty in EUROPE and AMERICA were under deeper obligations than to the Medical and Physical Journal of London, now forming a long, but an invaluable, series."—RUSH.

*For the London Medical and Physical Journal.*

### *On Diseases erroneously considered, and Treated as Syphilitic;* by Mr. RICHARD CARMICHAEL.

THE practice, in these countries, of resorting to the exhibition of mercury in every species of venereal disease, is so general, and so firmly established, that it cannot be resisted with any chance of success, except by marshalling a host of facts in evidence of the inutility and injurious consequences of this mode of treatment.

As I avow this to be the object of the following paper, the reader is not to complain of *ennui* in perusing a number of cases bearing a close resemblance to each other; and, if they demonstrate the justness of my views, and the propriety of my practice, I trust they will excuse the unavoidable dullness and uniformity of the detail.

Of the several venereal diseases confounded with syphilis, which I have witnessed since the publication of my Essay, I shall begin with that class which my increasing experience enables me to state is probably more generally disseminated than all the other classes of venereal diseases together, even including syphilis in the number. The poison to which I allude is capable of producing three different affections, viz. 1st, a superficial ulcer, without induration or elevated edges; 2d, a patchy excoriation of the glans and prepuce, attended with purulent discharge; and 3d, a gonorrhœa virulenta. My reasons for concluding that these affections arise from one and the same poison have been stated at large in my Essay: at present it is sufficient to mention, that my conclusion is grounded on the following facts. 1st. Two, and sometimes the three affections were found to exist together in a multiplicity of instances, in the same person, at the same time. 2d. They yield severally or collectively to the same mode of treatment. And 3d, Each produces the same identical train of constitutional symptoms, viz. a papular eruption, attended with more or less fever, which terminates in desquamation of the cuticle, but which usually again recurs at irregular intervals; a chronic inflammation of the fauces, accompanied with difficult deglution, and an excoriated appearance of the back of the pharynx, and pains which chiefly affect the larger joints, but not unfrequently also the head and chest.

In addition to the observations contained in my Essay on this class of venereal disease, my further experience has taught me that the following circumstances also characterise the poison under consideration. 1st. The buboes it produces are frequently attended with a chronic enlargement of the glands of the groin, which remain inactive till excited either to resolution or suppuration by the application of repeated blisters. Although these swellings often gradually subside, yet, in the majority of instances, they proceed to suppuration, and form abscesses, which readily heal after being punctured, or allowed to break spontaneously, but sometimes their healing is protracted, by the protrusion of the enlarged glands through the ulcerated integuments. Mercury, either internally or externally used, seems to be incapable of dispersing those buboes; and, after ulceration has occurred, it often excites the most obstinate and inveterate extension of the ulcer.—2d. Besides the inflammation of the throat already described, a chronic enlargement of the tonsils not unfrequently occurs, so extensive, at times, as nearly to close the passage. The tonsils, in these cases, exhibit an irregular raw surface, not amounting to ulceration; but each enlarged tonsil projects through the double arch of the palate, and appears as if it had ruptured by its enlargement its own proper coat.—3d. The poison, when affecting the constitution, also frequently excites inflammation and enlargement of the cervical, submaxillary, and parotid glands, which usually suppurate. These swellings have been hitherto either supposed to arise from cold, while the patient was under the influence of mercury, or else to a power attributed to that mineral of exciting into action scrofula, when latent in the constitution. These conjectures and hypotheses have, however, no foundation in truth, as the affection under consideration arises where mercury has not at all been exhibited.—4th. During the continuance of the papular eruption, chronic inflammation of the conjunctiva (totally distinct from syphilitic ophthalmia, which chiefly affects the iris, and also distinct from the acute inflammation of the conjunctiva attended with purulent discharge, which arises from the contact contact of the gonorrhœal virus) is a frequent attendant symptom. In this affection of the eyes, as well as in that of the throat, we recognise only analogous effects of the morbid poison under consideration, with all other morbid poisons which produce eruptions attended with fever; for instance, the small-pox, measles, and scarlatina.—5th. Nodes, or enlargement of the bones, so frequently a symptom of the poison of syphilis, and also of that which produces the phagedenic ulcers, are never produced by the poison under consideration. I feel warranted in drawing this conclusion, as I have not in any instance observed that the primary affections which are the subject of this paper were followed by nodes.

The reader will bear in mind that I am about to select from my case-books instances only of one class of the diseases which have been confounded with syphilis. These I shall detail according to the series in which they occurred; and, in order to prevent, as far as lies in my power, any cavil, I shall not bring forward any evidence from my private practice, but shall detail only those cases which occurred in Hospital, the authenticity of which cannot be doubted, for cases in public hospitals are necessarily under the superintendance of many, and open to the examination of such of the profession as chuse to attend to them. The admission and discharge of the patients are regularly registered, and the medicines prescribed recorded in the prescription books, a registry which remains as an incontrovertible document of the authenticity of the facts detailed.

The cases are so numerous, even in this single class of venereal diseases, and their progress and treatment so very similar, that I was at first inclined to give them to the public in a tabular form; but I soon perceived that in pursuing this plan, I should necessarily omit many useful facts. I shall, however, detail them in the briefest manner, and compress the statement of every necessary fact into the smallest possible compass. With this view, I beg to premise, that the letters R. H. are intended to signify that the patient was admitted into the Richmond Hospital. The other cases not marked with these initials are to be understood as treated in the Lock Hospital. The abbreviations Solut. Antim. signify the antimonial solution, prepared as follows:

℞. Tartari Antim. gr. iv, solve Aquæ ℥vij, et adde

Tinct. Opii ʒj.

Tinct. Cardamom. comp. ʒiij.

Syrupi Simpl. ℥ſs. M.

Of this solution the patient usually took a table-spoonful every fourth hour; but if it affected his stomach, but half of that quantity was prescribed. The abbreviations Lot. flav.

 signify signify the yellow wash, composed of the corrosive muriate of mercury and lime water, in the proportion of either half a grain, or a grain to an ounce. The abbreviations Lot. Nig. signify the black wash, composed of calomel and lime water, in the proportion of ten grains to an ounce. Such patients as were affected with phymosis and discharge from within the prepuce, were directed to inject these lotions, every second hour, between the glans and prepuce; and such as were affected with ulcers, were ordered to keep them constantly moistened with lint soaked in the lotion directed. The words Fotus and Cataplasma signify warm fomentations, composed of decoction of chamomile flowers and poultices of bread and water, which were employed in cases of inflammation, or of irritable painful ulcers of the penis, under which circumstances the patient was always confined to the recumbent position. Wherever the term discharge occurs in connection with phymosis, it implies a discharge of matter either from the excoriated surface of the glans and prepuce, or from ulcers; for, during the continuance of phymosis, it is in general impossible to ascertain from which of these sources the discharge proceeds.

Case I.—Daniel Ellis, November 16th, 1813. An ulcer, the size of sixpence, on the body of the penis; a smaller one on the external prepuce; attended with a fullness of the surrounding parts, pain in deglutition, and slight inflammation of the fauces; pains in his shoulders, elbows, hips, and knees. He stated that he was five weeks disordered; and that he took mercurial pills during three weeks, but that his complaints did not amend under their use. Decoct. Sarsap. Solut. Antim. Lot. flav. Dec. 6th, discharged the Hospital well.

Case II.—William Doolan, R. H., Dec. 7th, 1813. Phymosis, considerable inflammation and swelling of the penis, discharge. He stated that he had been sixteen days disordered, and had not used mercury. Venæsect. ad ℥xvi. Fotus-Catapl. Solut. Antim. Dec. 14th, inflammation removed. Lot. flav. Dec. 28th, discharged the Hospital well.

Case III.—John Curvin, Dec 8th, 1813. Phymosis, swelling and inflammation of the entire penis, discharge, gonorrhœa, ardor urinæ, considerable symptomatic fever, pulse 100. He stated that he had been a fortnight disordered; that he had not used mercury; and that the first complaint with which he was affected was the gonorrhœa. Venæsect. ad ℥xvi. Solut. Antim. Fotus Catapl. On the 20th, the inflammation was removed, and I directed him to inject frequently, both into the urethra and between the glans and prepuce, an injection composed of 20 grains of calomel to

8 ounces

8 ounces of lime water. On the 5th of January he was discharged the Hospital well.

Case IV.—Patrick Cosgrave, Dec. 8th, 1813. Phymosis, discharge, some swelling and inflammation of the penis, bubo containing matter. He stated that he had been five weeks disordered, and that he rubbed in five drachms of mercurial ointment. Fotus. Catapl. Solut. Antim. Dec. 13th, the inflammation and swelling removed; bubo had ulcerated. Solut. Antim. Lot. flav. Dec. 27th, discharged the Hospital well.

Case V.—John Mahon, Dec. 23d, 1813. Pariphymosis; a circle of sores round the corona glandis and adjoining surface of the prepuce. He stated that he had been disordered three months, and had used mercury. Solut. Antim. Catapl. Jan. 10th, he was discharged the Hospital well.

Case VI.—Jeremiah Hogan, Jan. 25, 1814. Phymosis; a large ulcerated opening in the superior part of the prepuce, which exposed part of the glans and corona, and gave passage to a profuse purulent discharge. He stated that he had been two months disordered; that the pain and inflammation were so severe as to cause a slough of that part of the prepuce which left the opening above mentioned; and that he had used mercury extensively. Solut. Antim. Lot. flav. Feb. 8th, discharged well.

Case VII.—Daniel Kinshela, Feb. 11th, 1814. Phymosis; several small sores on the urethral side of the body of the penis; and a considerable number of raised fungous looking sores on the scrotum, inside of the thighs, and fossa of the nates. There was also a small abscess, like a boil, the skin covering which was of a livid colour, situated on the inside of one of his thighs, near the groin. He stated that he had been disordered from the preceding July, a period of seven months; that he had been the greater part of that time kept under the influence of mercury, without any amendment of his complaints; that the first symptom with which he was affected was a profuse gonorrhœa; and that the sores occurred within the three months previous to his admission. The application made use of on this and other similar occasions was the frequent ablution of those fungous sores with a strong solution of corrosive muriate of mercury, in the proportion of half a drachm to eight ounces of water, which caused considerable smarting, but healed the ulcers before the 23d of February, in the short space of twelve days, when he was discharged the Hospital. The yellow wash was applied to the ulcers on the penis; and he took the antimonial solution.

Case VIII.—Michael Whelan, Feb. 14th, 1814. Excoriation of the glans and prepuce, with purulent discharge; small small superficial sores on the urethral side of the penis, and on the scrotum. Solut. Antim. Lot. flav. March 1st, discharged well.

Case IX.—John Byrne, Feb. 24th, 1814. Small superficial sores on the corona glandis. He stated that he had been a fortnight disordered, and had not used any mercury. Lot. flav. March 1st, discharged well.

Case X.—James Barry, Feb. 24th, 1814. An ulcer on the body of the penis, the size of a sixpence; excoriation of the anterior surface of the scrotum. Solut. Antim. Lot. flav. March 1st, discharged well.

Case XI.—George Enniss, March 7th, 1814. Gonorrhœa, paraphymosis, a small ulcer on the prepuce. He stated that he had been disordered a fortnight, had not used mercury, and that the paraphymosis occurred a week previous to his admission. Solut. Antim. Catapl. He was also desired to use an injection of one grain of muriate of mercury to six ounces of lime water, which was afterwards increased to two grains to six ounces of lime water, without exciting any pain. April 18th, complaints all well, discharged.

Case XII.—James Duff, March 7th, 1814. An eruption of papulæ, similar to that delineated in plate 2, fig. 5, of my Essay, covering almost every part of his body; pains in his shoulders, elbows, hips, and knees; throat inflamed; deglutition difficult; the eyes also red and inflamed, an appearance which usually occurs in a greater or less degree during the existence of the papular venereal eruption; considerable swellings of the sub-maxillary glands. He stated that the first appearance of the disorder was an ulcer on the prepuce, with which he was affected five weeks previous to his admission, and that it healed under the internal use of muriate of mercury, Decoct. Sarsap. Solut. Antim. Balneum tepidum. March 14th, his throat was well, but the pains were more severe. 21st, the eruption declining, and the pains were considerably lessened; the swelling of the glands of his neck were evidently suppurating. April 4th, the glands of his neck had ulcerated; a glandular swelling had also appeared in his right groin; the pains and eruption were daily declining. May 16th, discharged the Hospital, as he had no other complaints except the glandular tumours and ulcers on his neck.

Case XIII.—Henry Owens, March 10th, 1814. Several small ulcers on the fremum, and adjoining surface of the glans, prepuce, and corona. He stated that he had been nine weeks disordered, and took mercurial pills during that time. Solut. Antim. Lot. flav. April 4th, discharged well.

Case XIV.—William Ferrall, March 10th, 1814. A chain of of small ulcers around the corona, two or three on the internal surface of the prepuce; gonorrhœa; enlargement of the tonsils, attended with an irregularly ulcerated surface, but clean, and apparently granulating. He stated that he had been disordered five or six months; that the first sores on the penis spontaneously healed, but that they were followed by a succession of others, which healed in the same manner; that his throat became sore two months previous to his admission; and that he had not used mercury. Decoct. Sarsap. Solut. Antim. Lot. flav. March 22d, the ulcers of the penis healed, his throat improved, but he complained of pains in his shoulders and knees. April 11th, dyspnœa, and pain in his side, which rendered venæsection necessary: this symptom is by no means unfrequent in the disease under consideration. April 20th, discharged well.

Case XV.—Charles Kenny, March 22d, 1814. Small fungous smooth ulcers on the scrotum and fossa of the nates; considerable enlargement, and superficial ulcerated appearance, of the tonsils; deglutition difficult; hoarseness. He stated that he had been five months disordered, and had not used mercury. Solut. Antim. Lot. flav. April 4th, the ulcer of the scrotum and fossa of the nates healed. The ulcers with which this man was affected are usually considered constitutional symptoms of syphilis, and treated by a full course of mercury: I have always found that they yielded to the simple means employed in the present instance. April 20th, his throat was considerably improved, but the tonsils were still very much enlarged: contrary to my advice, he left the Hospital, but on the 8th of the following month was admitted. The tonsils at this time were so much enlarged as nearly to close the passage, and their surface was irregular and ulcerated. He also was affected with frequent spitting of blood, the source of which was not very obvious. Chiefly on account of the latter symptom, I put him on the use of nitrous acid, and directed him to take fifteen drops of the tincture of digitalis thrice a day. May 23d, the spitting of blood had ceased; the appearances in his throat were unchanged. Decoct. Sarsap. Solut. Antim. June 6th, the ulceration of the tonsils were healed, and the swelling diminished. June 13th, the swelling of the tonsils was daily lessening; discharged the Hospital.

Case XVI.—Thomas Finn, April 8th, 1814. Excoriation of the glans and prepuce; bubo in the left groin. He stated that he had been a month disordered, and had not used mercury. Lot. flav. Solut. Antim. April 11th, the excoriation healed; bubo coming forward. April 25th, bubo had ulcerated, and exhibited a deep cavity, with loose undermined edges, which

I directed to be touched daily with caustic. May 31st, discharged the hospital.

Case XVII.—William Flinn, April 14th, 1814. A superficial whitish sore surrounding the orifice of the urethra; excoriated patches, discharging matter, on the scrotum and fossa of the nates; copper-coloured spots on his forehead, breast, and back, which, on a superficial view, might be taken for the psoriasis syphilitica, but, on a close examination, was decidedly the papular eruption in its advanced or desquamating stage. He stated that he had been two months disordered, and had not used any mercury; and that the eruption appeared a fortnight previous to his admission, from which we learn, that the constitutional symptoms may appear in the species of venereal disease under consideration six weeks after infection, and that the eruption will have far advanced in desquamation a fortnight after its commencement. Decoct. Sarsap. Solut. Antim. Lot. flav. April 25th, all the ulcers had healed, and the eruption had entirely disappeared, leaving the skin slightly discoloured. May 2d, discharged well.

Case XVIII.—Patrick Hoey, April 21st, 1814. Small ulcers on the prepuce and scrotum; indolent bubo; right groin with a small opening discharging matter. He stated that he was disordered the preceding September; and that he took mercurial pills, which affected his mouth. Solut. Antim. Lot. flav. May 2d, ulcers all healed; discharged.

Case XIX.—Edward Malone, May 2d, 1814. Phymosis, with high inflammation; discharge; pulse 96. He stated that he had been a fortnight disordered, and had not used mercury. Venæsect. ad ℥xvi. Solut. Antim. Fotus. Catapl. May 9th, the superior part of the prepuce, to the extent of a shilling, had sloughed; inflammation gone. Lot. flav. May 22d, the discharge stopped; ulcer healed; an eruption of large yellow pustules had appeared on his hands and wrists, which declined before the 31st, on which day he was discharged.

Case XX.—Charles Hannagau, May 19th, 1814. An extensive ulcer, of a rough unequal surface, and without induration, engaging the frenum, inferior surface of the glans penis, orifice of the urethra, and a considerable portion of the prepuce. It had not the characters of chancre, or of a phagedenic ulcer, neither had it the usual appearance of ulcers described in this paper: its surface, which was of a healthy colour, was irregularly intesected by raised fleshy bands, bearing somewhat a resemblance to the internal surface of the ventricles of the heart. I am thus minute in my description, because I have met, in private practice, two similar cases of ulceration, which resisted full courses of mercury, cury, were not checked in their progress until that medicine was laid aside, and recourse was had to emollient poultices and fomentations, together with the internal use of antimonials. In the present instance, the patient had undergone, before I saw him, a severe course of mercury, which did not produce any beneficial change. I directed the antimonial solution, decoction of sarsaparilla, and poultices of bread and water, under which the ulcer slowly but gradually improved, and was healed before the 18th of July, on which day he was discharged the hospital well.

Case XXI.—Matthew Simpson, May 30, 1814. Papular eruption almost covering his face and back, but thinly scattered over his breast and belly. It nearly resembled that delineated in plate 2, fig. 6, of my Essay. He complained of pains in his shoulders, elbows, knees, and insteps; chronic inflammation of the fauces, with difficult deglution; pulse 90. He stated, that six weeks previous to his admission, he had been affected with an ulcer on the prepuce, which healed spontaneously; and that three weeks afterwards the eruption appeared, for which he took a few mercurial pills. Solut. Antim. Decoct. Sarsap. June 26th, discharged the hospital well.

Case XXII.—Hugh Fitzpatrick, June 3, 1814. Phymosis; discharge; small ulcers were apparent within the orifice of the prepuce. He stated that he had been disordered six weeks, and had not used mercury. Solut. Antim. Lot. Niger. June 13th, discharged well.

Case XXIII.—Edward Butler, June 6, 1814. Excoriation of the glans and prepuce. He stated that he had been disordered five weeks, and had not used mercury. Solut. Antim. Lot. flav. June 14th, discharged well.

Case XXIV. Richard Walsh, July 19, 1814. Phymosis; discharge; ulcers, of smooth surface, were apparent within the orifice of the prepuce; gonorrhœa; ardor urinæ; bubo. He complained of pains in his shoulders; and stated that he had been two months disordered, and had not used mercury. Solut. Antim. Lot. flav. July 25th, an eruption of papulæ had appeared on his face and arms; the pains of his shoulders were more severe; he also complained of pain in the right knee, particularly at night. Decoct. Sarsap. Solut. Antim. Lot. flav. These pains continued severe until the 8th of August, at which time they, as well as the eruption, began to decline. The bubo had suppurated, but all his complaints were removed before the 26th of August, on which day he was discharged the hospital well.

Case XXV.—John Lewis, *Sept. 20, 1814. An extensive

* Such cases as occurred between this and the preceding date were not noted, in consequence of my absence from the hospital.

granulating ulcer, of an irregular surface, and interspersed with warts, engaged the greater portion of the prepuce and glans; there was a large opening in the prepuce, probably caused by a slough, through which the glans protruded; the entire penis, but particularly the prepuce, was much swollen; bubo, right groin. He stated that he had been disordered since the preceding January, a period of eight months, and that he had been using mercury the greater part of that time. Solut. Antim. Catapl. Oct. 3d, the ulcer in the same state, but painful. Capt. Ext. Cicutæ gr. v. ter in die. Oct. 6th, the dose of cicuta was increased to ten grains, and a solution of opium was added to the poultice. Oct. 10th, the ulcer looked worse, and became extremely painful, attended with inflammation of the entire penis, and considerable symptomatic fever, which was partly caused by the bubo, which had become large and inflamed. Omitt. Cicut. et Capt. Solut. Antim. Venæsect. ad ℥xvi. Fotus. Catapl. Oct. 17th, the pain and inflammation were reduced; many large papulæ had appeared on his belly and thighs. Omit. Solut. Antim. et Catapl. Capt. Ext. Cicutæ gr. v. ter die. Decoct. Sarsap. Lot. flav. From this period the ulcer continued slowly and gradually to improve, but was not healed until the 5th of December. When the irritable state of the ulcer was subdued, the acetic acid was applied to the warts, which were destroyed by it in a short time. The obstinacy of this ulcer I attributed to the great irritation produced by these warts; and, with a view chiefly to alleviation, the cicuta was employed. On the 12th Dec. he was discharged well.

Case XXVI.—Abraham Warner, Sept. 20, 1814. Partial phymosis; an ulcer on the inferior portion of the glans; discharge; papular eruption on every part of his body; pains in his shoulders, elbows, knees, and ancles. He stated that he had been disordered five months, and had taken a great number of mercurial pills. Decoct. Sarsap. Solut. Antim. Lot. flav. Sep. 26th, the ulcer had healed, and the glans could be completely denuded, which exposed a large collection of warts on the corona: to these the acetic acid was daily applied. The eruption and pains were declining. Oct. 31st, discharged well.

Case XXVII.—William Stoyte, Oct. 10, 1814. Phymosis; swelling and violent inflammation of the entire penis; hæmorrhage from beneath the prepuce, the superior part of which, to the extent of a shilling, was black and mortified; pulse 120; thirst, and other signs of symptomatic fever. He stated, that ten days before he observed an ulcer on the glans, to which he applied an irritating wash, and to this circumstance, and want of care, he attributed the inflammation.

tion. He also mentioned, that on the morning of his admission, he lost at least three pints of blood. Fotus. Catapl. Solut. Antim. The following day the slough of the prepuce had separated, by which the glans was exposed, and he felt himself easier. Oct. 12th, the pain had returned with great violence; pulse 120. A cathartic mixture was directed, containing tartarized antimony. Venæsect. ad ℥xvi. On the day following, the remainder of the prepuce was found in a state of slough; pain not relieved. Rept. Mist. Cathart. et Venæsect. ut heri. Habeat haust. Anody. h.s. Oct. 15th, the pain, inflammation, and symptomatic fever reduced. Opii gr. ij. o. n. Oct. 18th, the ulcer was considerably amended. Solut. Antim. Pil. Opii ut antea. From this period his amendment was decided but slow. The ulcer, which had destroyed the entire prepuce, and a great portion of the glans, was healed before the 12th of December, on which day he was discharged the hospital well.

Case XXVIII.—John Day, Nov. 1, 1814. An extensive ulcer of the corona glandis; bubo containing matter in each groin, which I directed to be poulticed. He stated that he had been two months disordered, and had not used mercury. Solut. Antim. Lot. flav. Dec. 1st, the ulcer of the penis had healed; the buboes had ulcerated. Dec. 6th, discharged well.

Case XXIX.—Richard Thompson, Nov. 24, 1814. Small ulcer of the corona and prepuce; excoriation; discharge. He stated that he had been three months disordered, and had not used mercury. Solut. Antim. Lot. flav. Dec. 5th, the ulcers and excoriation had healed, but he complained of pains in his shins and thighs. Dec. 19th, the pains had left him, and he was discharged the hospital. Dec. 26th, he was re-admitted on account of a return of ulceration on the corona, and of pains which he complained of in his nose and arms. Decoct. Sarsap. Solut. Antim. He gradually amended, and was discharged the hospital well on the 29th January, 1815.

Case XXX.—Hugh Brogan, R. H. Nov. 24, 1814. Three small ulcers on the corona glandis; bubo right groin; he had not used mercury. Solut. Antim. Lot. flav. Dec. 20th, discharged well. It was from this man the infection was taken with which Phelan and Dunn were inoculated, whose cases are detailed in p. 189 and seq. of my Essay.

Case XXXI.—Michael Corry, R. H. Nov. 24, 1814. A smooth fungous-looking ulcer on the frenum and adjoining parts; bubo left groin. He stated that he had been four months disordered, and had undergone a course of mercury of six weeks duration. I directed that the ulcer should be touched with lunar caustic daily. Solut. Antim. Lot. flav.

 Dec.

Dec. 20th, the ulcer was healed; the bubo, which was small, and contained matter, was punctured. Dec. 26th, the bubo had nearly healed; discharged.

Case XXXII.—George Hutchinson, R. H. Nov. 24, 1814. Phymosis; inflammation of the entire penis; discharge. He stated that he had been six weeks disordered, and had undergone a course of mercurial frictions. Venæsect. ad ℥xvi. Solut. Antim. Fotus. Catapl. Dec. 5th, the inflammation and swelling were reduced, but he was attacked with severe pains in all his joints, constant nausea and vomiting, thirst and general fever; pulse 110; tongue white and furred. Venæsect. ad ℥xvi. Mistura effervescens sæpe in die. During this attack of fever and pains in the joints, which continued violent until the 20th of December, he took saline and other aperient medicines, and also u-ed the yellow wash frequently, which stopped the discharge. On the 26th Dec. he was attacked again with fever and severe pains in the joints, for which he underwent the same treatment as before; but he did not recover perfectly before the 20th Jan. 1815, on which day he was discharged the hospital. Many will be inclined to imagine that the acute fever* and pains that occurred in this instance were unconnected with the venereal virus; but I have so frequently witnessed fever and pains, equally acute, in the class of venereal diseases under consideration, many cases of which were at the same time accompanied by the papular eruption, that I had no hesitation in considering those symptoms as constitutional effects of the poison.

Case XXXIII.—Patrick Ward, R. H. Nov. 28, 1814. An extensive ulcer, which engaged one side of the glans penis, and the adjoining surface of the prepuce, which was swelled and inflamed. That part of the ulcer situated on the glans exhibited the characters of the species of venereal ulcer under consideration; but that situated on the prepuce looked foul, and was almost phagedenic in appearance, which I attributed to the inflammation of the prepuce. He stated that he had been ten weeks disordered, and had taken but five mercurial pills. Solut. Antim. Fotus. Catapl. Dec. 20th, that part of the ulcer situated on the glans was healing, but that on the prepuce was but little improved. Omitt. Solut. Antim. et Capt. Ext. Cicutæ gr. v. ter die. Lot. flav. Jan. 2, 1815, the ulcer was improved. Ext. Cicutæ gr. x. ter die. Jan. 16th, discharged well.

Case XXXIV.—James Stewart, R. H. Dec. 18, 1814. Phymosis; profuse discharge; bubo. He stated that he had

* Dr. Adams considers fever during the *primary* symptoms of some morbid poisons mistaken for syphilis, as among the distinguishing marks. See Mordid Poisons, 2d edit. p. 47.—Edit.

been been three weeks disordered, and had not used mercury. Solut. Antim. Lot. flav. Jan. 16, 1815, the discharge was almost stopped, and he could retract the prepuce, which exposed a superficial ulcer of the glans, and the cicatrices of several that had healed; but he complained of pains in his shoulders and arms, which, however, soon left him, and, the ulcer having healed, he was discharged on the 24th January.

Case XXXV.—Thomas Gibbons, Dec. 20, 1814. Pain of the left knee and leg, and severe pains in both his heels, which almost prevented him from walking. He stated that he never had, at any time, ulcers on the genitals, but that several times he was affected with gonorrhœa; the last occurred four years previous to his admission: that in 1811 he underwent a severe mercurial course, on account of similar pains, without obtaining any relief; and that they had continued from that time to the present. Decoct. Rament. Ligui Guaici* ℔j. quotidie. Pulv. Ipecac. comp. gr. x. ter in die. As he did not derive any benefit from these medicines, I put him, on the 9th Jan. 1815, upon a full mercurial course, both by mercurial pills and inunction, which he persevered in till the 6th of March, during which period, a strong mercurial action was preserved in his system, with no other effect than alleviation of the pain of his knee and leg: the pains of his heels, of which he chiefly complained, were not in the slightest degree amended by the mercury. The decoction of guaicum was again resorted to, with no greater success than before. On the 20th March I resolved to try the effects of counter-irritation, and directed successive blisters to be applied on the heels, extending from one ankle to the other. This plan proved more successful than the others: alleviation soon followed its adoption, and the pains were totally subdued before the 24th of April, on which day he was discharged the hospital well. This case, as well as many others I have noted, affords an instance of the removal of obstinate pains by counter-irritation, which mercurial courses were found incapable of subduing. I know not the quantity of mercury this man used in Stephens' Hospital, but under my care, independant of pills, he rubbed in four ounces of ointment. Instead of blisters, I frequently employ the ointment of tartarized antimony, which produces a greater degree of irritation.

Case XXXVI.—John Culbert, Dec. 28, 1814. Phymosis; discharge; large smooth ulcer external prepuce; bubo. He stated that he had been five months disordered, and that

* ℞. Lig. Rament. Guaici ℥ ij. Aquæ ℔ij. Decoque ad ℔j. et Cola.

he he had used mercury extensively. Solut. Antim. Lot. flav. Feb. 5, 1815, the ulcer healed, and the discharge was stopped: an eruption of papulæ had, however, appeared on his face, back, and breast, attended with difficult deglutition, inflammatory appearance of the fauces, and pains in the wrists. 20th, the pains were more general, and affected his shoulders and elbows; the eruption was declining. Decoct. Sarsap. Solut. Antim. 27th, the eruption had disappeared; the pains were subdued; and he was discharged the hospital. He was, however, re-admitted on the 20th of April, on account of an eruption of very small papulæ collected into distinct separate patches, which were scattered over every part of his body, but were particularly numerous on his face. His throat was affected in the same manner as before; his eyes were red and inflamed; and he complained of pains in all the larger joints: there was also phymosis, with a slight discharge. Solut. Antim. Lot. flav. May 1st, he complained that the pains of his head and joints were more severe: I directed a pill containing 3 grs. of antimonial powder and gr. ſs. of calomel to be taken three times a day, and the antimonial solution to be discontinued. These pills affected his mouth slightly; and, under their influence, both the pains and eruption gradually declined, and he was discharged well on the 5th June. When the pains are obstinate, and unattended by fever, calomel in alterative doses, combined with antimony, has been found, in every instance, sufficient for their removal.

Case XXXVII.—Thomas Graham, ℞. H. Dec. 29, 1814. Phymosis; discharge; ulcerated bubo; small ulcers were apparent near the orifice of the prepuce. He stated that he had been a month disordered, and had not used mercury. Solut. Antim. Lot. flav. Feb. 7th, discharged well.

Case XXXVIII.—William Turner, R. H. Dec. 29, 1814. Phymosis; ulcers on the external prepuce; discharge; bubo. He stated that he had been three months disordered, and had not used mercury. Jan. 10th, the ulcer on the prepuce healed; he was able to retract the prepuce, which exposed a large smooth ulcer on the glans. 16th, he complained of pains in his shoulders, attended by the papular eruption; the ulcer of the glans was nearly healed. Jan. 21st, discharged well.

Case XXXIX.—Dan. Dempsey, R.H. Jan. 2, 1815. Phymosis, attended with acute inflammation; discharge; bubo. He stated that he had been affected with an ulcer on the glans two months previous to his admission, for which he had taken a considerable number of mercurial pills. Venæsect. ad ℥xvi. Solut. Antim. Fotus. Catapl. Jan. 9th, the inflammation flammation was reduced; the bubo had ulcerated. January 29th, discharged well.

Case XL.—Thomas Martin, January 3, 1815. Phymosis, great inflammation and swelling of the penis, severe pain, profuse discharge, bubo; pulse 100. He stated that he observed an ulcer on the prepuce three weeks before, that he had not used mercury, but continued at his employment. Venesect ad ℥xvi., Solut. Antim., Fotus. Catapl. January 16th, the swelling and inflammation had subsided; the bubo large, hard, and indolent. To this I directed a succession of blisters. Solut. Antim. Lot. flav. The bubo was dispersed by repeated blistering. On the reduction of the phymosis, ulcers on the glans and prepuce were exposed, which soon healed, and he was discharged the hospital on the 20th of February.

Case XLI.—John Murphy, January 18, 1815, R. H. Excoriation of the corona, discharge, partial phymosis, papular eruption, pains in his shoulders. Solut. Antim. Lot. flav. February 7th, discharged well.

Case XLII.—Patrick Tierney, R. H. January 24, 1815. Partial phymosis and inflammatory swelling of the penis, ulcer on the frenum and adjoining surface, bubo right groin. He stated that he had been five months disordered, and using mercury in an irregular manner the greater part of that time. Solut. Antim. Lot. flav. February 7th, he was able to retract the prepuce which exposed a number of pendulous warts, the largest of these were removed with scissars, and the remainder by the application of Acetic Acid. February 17th, discharged well.

Case XLIII.—Owen Clarke, R. H. January 25, 1815. The entire prepuce was, at the time of his admission, in a state of slough, attended with high inflammation of the entire penis, and acute symptomatic fever. Pulse 120. Mittet. Sanguis ad ℥xvi. Solut. Antim. Fotus. Catapl. The greater part of the slough was removed with scissars. 29th, the ulcer looked clean, and granulations were apparent, a proof that it was not of the phagedenic species. The ulcer engaged the entire corona, and a considerable portion of the glans, it also committed ravages on the body of the penis. February 12th, the ulcer was healing, and on the 21st was perfectly cicatrized. Discharged.

Case XLIV.—William Barry, February 2, 1815. Phymosis, profuse discharge, general enlargement of the penis from continued inflammation; several small spots covered with crusts on his groins and the upper part of the thighs. As there were not similar spots on any other part of his body, I concluded that these were owing to the local application of the the discharge from the penis, which was unusually great, and which, from the patients inattention to cleanliness, was allowed to contaminate the adjoining surface. These spots, I therefore inferred, were primary, and not constitutional ulcers. He stated that he had been disordered eight weeks; that previous to the attack of phymosis, several ulcers were apparent on the glans and internal surface of the prepuce, and that he had not used mercury. Solut. Antim. Lot. fl. 13th, the swelling and discharge were lessened. 26th, the discharge had ceased, and he was able to retract the prepuce which exposed several fresh cicatrixes. The crusts had separated from the spots on the thighs, leaving the ulcers underneath nearly healed. March 6th, discharged well.

Case XLV.—Michael Crawley, R. H. February 6, 1815. Phymosis, inflammation of the entire penis; discharge, bubo, quick pulse, thirst, and general fever. He stated that he had been a fortnight disordered, and that he had taken pills which affected his mouth. Solut. Antim. Fotus. Catapl. 16th, the inflammation and discharge lessened, the bubo dispersed. Solut. Antim. Lot. fl. February 21st, the discharge and phymosis entirely removed, discharged from the hospital.

Case XLVI.—Christopher Doyle, February 16, 1815. Phymosis, discharge; an extensive superficial ulcer which nearly engaged the entire surface of the body of the penis, a general eruption of papula in their different stages, some at their commencement, others arrived at maturity, and others terminating in disquamation of the cuticle. With these the entire surface of his body was nearly covered. On his hands the eruption closely resembled that of the common itch; he complained of difficulty of swallowing, and the fauces on examination appeared raw and inflamed. He stated that he had been five months disordered, that three months previous to his admission the eruption appeared, and that he had not used mercury in any form. Lot. fl. 27th, two-thirds of the ulcer were healed, discharge lessened, the eruption declining. He complained of pains in his knees. March 10th, the ulcer healed, the eruption continued to decline, pains in his joints more general. The Antim. Solut. and Decoct. of Sarsap. were discontinued, and he was ordered Decoction of Guaicum Wood. 27th, the ulcer was healed, the pains no longer felt, and the eruption was declining, but the papulæ on the back of his neck uniting with each other formed thin moist scales, which were very itchy and troublesome. On this part he was desired to smear an ointment composed of equal parts of tar and sulphur ointments. This application was attended with the desired effect. The eruption soon disappeared, and he was discharged the hospital on the 24th of April; but, on the 13th of the following July, he was again admitted on account of a fresh crop of the eruption, which, however, was not so general as before, and which he stated to have appeared a month after he had left the hospital. It exhibited papula in all their stages. There was also an ulcer almost the size of half-a-crown on the inner ankle of one leg, which was probably owing to the irritation and rubbing of this part while covered by the papula. He was directed to take a pill thrice a day, containing gr. ſs. of Calomel, and three grains of Antimonial powder. The ulcer was poulticed with bread and water. August 7th, he was discharged the hospital well.

Case XLVII.—James Martin, R. H. February 27, 1815. An ulcer engaging the frenum and adjoining surface of the glans and prepuce; an incipient bubo on the left groin. He complained of pain in his shoulders and knees, and stated that he had been five months disordered, and had not used mercury. In order to ascertain if mercurial frictions were capable of dispersing a bubo arising from this species of ulcer, I directed him to rub in a dram of mercurial ointment every night, as it was apparently in a favourable state for resolution, not indicating any signs of suppuration. March 20th, his mouth was strongly affected; the ulcer of the penis had healed, but the bubo had advanced to suppuration and discharged a large quantity of matter; the pains of his shoulders, back, and knees, continued more severe than before, so that the trial I had made of mercury in this instance, served to prove that it was not only incapable of impeding the progress of the bubo to suppuration, but also to demonstrate its inefficacy when opposed to the pain which attend this species of venereal disorder. 24th, the bubo had healed, and, as the pains still continued, notwithstanding that he still persevered in the use of mercury, I laid that medicine aside, and put him on the use of decoction of Sarsaparilla and Antimonial Solution. Under these medicines he derived immediate relief, and was discharged well on the 4th of April.

Case XLVIII.—James Smyth, March 13, 1815. Phymosis, discharge, bubo. He stated that he had been two months disordered, and had not used mercury. Solut. Antim. Lot. fl. April 17th, discharged well.

Case XLIX.—Luke Pew, R. H. March 14, 1815. Phymosis, discharge, gonorrhœa, ardor urinæ. He stated that he had been three weeks disordered and had not used mercury. Solut. Antim. Lot. fl. 16th, I found him affected with periphimosis in consequence of his forcibly retracting the prepuce, puce, which exposed two superficial ulcers on the glans. The periphimosis was reduced, and he was desired to persevere as before. 20th, the phymosis was removed, and the ulcers of the glans already healed. The gonorrhoea and ardor urinæ were unabated, for these he was directed to use an injection composed of gr. ſs. of Muriate of Mercury to ℥ vi. of lime water. April 4th, the running and ardor urinæ were lessened. He was discharged the hospital, but desired to persevere in the injection.

Case L.—Elizabeth Mara, R. H. March 18, 1815. An eruption on her legs, thighs, and arms, of papulæ in their declining or desquamating stage; in this state they had a close resemblance to the psoriasis syphilitica. She stated that she was a married woman, had been six months disordered, that she never perceived any ulcer or discharge on the pudenda, but that the first symptom with which she was affected was a soreness in her throat followed by the eruption above described, and pains in her head and different joints. For these complaints she was admitted into an hospital in this city, where she remained eight weeks, during which time she underwent a severe course of mercury which had no effect whatever on her complaints, and she was discharged the hospital for the benefit of pure air. Decoct. Sarsap. Solut. Antim. March 21st, the eruption was gradually declining, and she was discharged the hospital on the 28th of March, well.

Case LI.—John Fenning, R. H. April 1, 1815. Excoriation of the glans and prepuce, large indolent bubo right groin; complained of pains in his shoulders, ears, and ankles; and stated that he had been four months disordered, and had not used mercury. Solut. Antim. Lot. fl. April 14th, the excoriation healed; the bubo increasing in size, but not indicating any signs of suppuration. From this period to the 30th, he was affected with considerable fever, thirst, quick pulse, constipated bowels, and pains in his joints, for which antimonials and frequent purgatives were directed. The bubo continued to enlarge, and at length indicating signs of matter, was opened by a deep puncture on the 17th of May, and a small quantity of matter discharged which did not diminish its size. He was directed to take a pill thrice a day, containing three grains of Antimonial powder and half a grain of Calomel. May 28th, all discharge from the bubo had ceased, but the swelling remained undiminished. Repeated blisters were directed to be applied for the purpose of exciting it into some kind of action, and the pills were continued. Under this treatment the indolent and inactive tumour gradually diminished; the lingering pains of his joints joints were removed, and he was discharged the hospital on the 10th of June, on account of irregular conduct, when he was nearly but not altogether well.

Case LII.—John Flinn, April 5, 1815. An ulcerated bubo, with high edges and large flabby granulations. He stated that two months previous to his admission, he had been affected with an ulcer on the internal surface of the prepuce, which healed without mercury, and that the bubo appeared three weeks after infection. Solut. Antim. Lot. fl. April 10th, an eruption of papulæ appeared on his breast and belly. April 17th, the papulæ which were not numerous had nearly declined; the bubo was healed, and he was discharged the hospital well.

Case LIII.—William Taylor, April 10, 1815. Excoriation of the glans and prepuce, gonorrhœa, ardor urinæ, bubo in each groin. He stated that he had been disordered six weeks, and had not used mercury. Solut. Antim. Lot. fl. 24th, the excoriation healed; the buboes were enlarging, but did not indicate any signs of suppuration; gonorrhœa in the same state. He was desired to use an injection composed of one grain of Muriate of Mercury to six ounces of lime water, and to rub the ointment of Tartarized Antimony on the buboes every night, with the view of exciting them by the stimulus of this application from their indolent to a more active state. 30th, the ointment occasioned an eruption of pustules over the surface of the buboes, which were evidently diminished. May 8th, the gonorrhœa ceased, and the buboes were nearly dispersed; discharged the hospital.

Case LIV.—John M'Giff, April 27, 1815. Papular eruption on his back, shoulders, breast, and face; the papulæ were small, and in their declining state, and clustered together into distinct irregular patches; the glands on both sides of the neck were swelled and tending to suppuration. He stated that the commencement of the disorder was a large ulcer on the penis, the cicatrix of which on examination, I found to be situated on the corona and prepuce, and without induration. He also stated that the eruption and swellings of his neck appeared together, a month previous to his admission. He positively asserted that he had not used mercury in any form, and that the ulcer spontaneously healed. The neglect of their complaints so common in the generality of persons who seek relief in hospitals, has, however, this advantage, that it enables us, in discriminating the symptoms, to assign each to its proper cause. The swelling of the cervical glands has hitherto been attributed either to exposure to cold, while the patient was under the influence of mercury, or to latent scrofula in the system, excited into action

 by by that medicine, but in the present as well as in many other similar cases which have occurred to me, no mercury had been taken, therefore those swellings can be properly attributed only to the constitutional effects of the poison. The medicines directed for this patient were as usual Sarsaparilla and Antimony. May 3d, the eruption was declining; the tumours of his neck were suppurating and he was desired to poultice them, he complained of pain in one of his knees. 15th, the eruption had disappeared and the pain of his knee was less acute; the abscess on each side of his neck was punctured. 22d, all his complaints were well except the sores attending the glandular swellings of his neck, and they were mending. Discharged the hospital.

Case LV.—John Twyfort, April 27, 1815. Inflamed and excoriated appearance of the fauces with difficult deglutition; pain and soreness upon pressure of one side of his head. He stated that five months previous to his admission he had been affected with an ulcer on the penis, for which he underwent a slight mercurial course in the Lock Hospital, and the ulcer healed; that three months afterwards, his present symptoms occurred, and also a severe pain in the ball of one of his eyes, which he said was attended with loss of vision in the affected eye, but which had been restored without the aid of medicine. Decoct. Sarsap. Solut. Antim. May 5th, his complaints were all well and he was discharged the hospital.

Case LVI.—Richard Moorhouse, April 24, 1815. Excoriation of the glans, corona, and prepuce; a papular eruption thinly scattered over his entire body; on his hands the spots were more pustular, and resembled those of the itch; two moist ulcerations of the skin in the neighbourhood of the anus, such as are usually considered indubitable signs of constitutional syphilis. They seem, however, to be produced in any species of venereal disease when the eruption occurs, on one portion of skin opposed by another, as in the fossa of the nates, axilla, and between the folds of the thighs, &c. He was also affected with chronic inflammation of the fauces, a difficulty of swallowing, and complained of pain in his thighs and legs, and in one of his knees. He stated that he had been ten months disordered, for which he used mercury repeatedly and extensively, but never under confinement. Solut. Antim. Lot. fl. The lotion he was desired to apply to the moist tumours in the neighbourhood of the anus as well as to the excoriation of the penis. The excoriation and the tumours were both removed before the 8th of May, and his other complaints disappearing, he was discharged the hospital on the 22d of the same month.

Case LVII.—John Dunn, May 18, 1815. A small superficial ficial ulcer on the prepuce, with a surrounding fullness, but not that callous induration which characterizes a chancre; a small ulcerated bubo in the right groin. He stated that he had been six weeks disordered, and had taken a few mercurial pills, but not sufficient to affect his mouth. Solut. Antim. Lot. fl. 29th, the ulcers were healing; another bubo had commenced in the left groin. June 13th, the ulcers of the penis and groin healed, the bubo of the left groin dispersed; discharged the hospital.

Case LVIII.—David Hays, May 18, 1815. Ulcer on the prepuce, excoriation of the corona glandis; bubo right groin; papular eruption scattered thickly over the entire body; the greater part of the papulæ were in their declining state; the glands of the upper part of his neck considerably enlarged. He complained of pains in his shoulders, elbows, knees, insteps, and chest; and stated that he had been two months disordered, that the eruption appeared three weeks after infection, and the swelling of his neck a short time previous to his admission, and that he had not used mercury. Decoct. Sarsap. Solut. Antim. 29th, the ulcer and excoriation of the penis healed; the pains were less severe, and the eruption was declining. June 5th, the bubo which had considerably enlarged was hard and indolent, and a second had appeared in the left groin; to these tumours I directed the application of repeated blisters. 26th, the bubo of the right groin still hard and indolent, that of the left groin had suppurated and was freely opened. The tumours of his neck had also enlarged, and one over the carotid gland containing matter was punctured; another beneath the right ear had ulcerated. July 11th, a fresh crop of papulæ of a large size had appeared on both legs, attended with pains in his elbows and insteps. The indolent bubo to which successive blisters were applied was gradually diminishing in size; the other was nearly healed. He was desired to keep lint wet in a solution of Sulphat of Zinc (ʒi. to ℔i.) constantly to the ulcers and tumours of his neck. July 23d, the papulæ on his legs were so large, that in their declining or desquamating state they formed their scales or crusts, which, however, had not ulcers beneath them, as is the case in the pustular eruption of the phagedenic ulcer. The tumours of his neck were diminishing and the ulcers healing, the pains were also declinng. August 7th, discharged well.

Case LIX.—Francis Brady, R. H. May 22, 1815. Phymosis discharge, inflammation and swelling of the entire penis. He stated that he had been six weeks disordered previous to his admission, and that he took a few pills, which did not affect his mouth. Venæsect ad ℥xvi.; Solut. Antim. Fotus.

Fotus. Catapl. June 1st, inflammation reduced. Lot. flav. 8th, nearly well, discharged at his own request.

Case LX.—James M'Menus, R. H. May 23, 1815. Phymosis, general enlargement of the penis, a superficial ulcer which extended from the pubes to the orifice of the prepuce engaging the dorsum and sides of the penis; two small fungous looking ulcers on the inside of the left thigh. He stated that he had been three months disordered and had taken a few pills, which did not affect his mouth. Solut. Antim., Fotus. Catapl. The inflammation lessened; ulcer improved. Lot. fl. June 18th, the ulcers of his thighs healed; that of the penis nearly cicatrised; an eruption of papulæ had appeared on his breast and arms. July 12th, the papulæ continued to appear in succession over the entire body, attended with chronic inflammation of the fauces, and swelling of the cervical glands; the ulcer of the penis had healed; discharged on account of irregularity.

Case LXI.—Thomas Kelly, May 25, 1815. An ulcer on the side of the body of the penis slightly excoriated and without induration; an incipient bubo on each groin. Solut. Antim., Lot. fl. 29th, the ulcer considerably improved and exhibiting more decidedly the characters of the smooth superficial ulcer. June 13th, the ulcer healed, buboes dispersed; discharged from the hospital.

Case LXII.—James Kelly, R. H. June 6, 1815. Gonorrhœa, ardor urinæ, ulcerated bubo in the left groin, swelling of the testis. He stated that he had been three months disordered; that he had ulcers on the penis, which healed spontaneously, and that he had taken a few mercurial pills afterwards which did not affect his mouth. Solut. Antim. Fotus. Catapl. July 17th, bubo healed, swelling of the testis reduced, gonorrhœa diminished. Discharged the hospital.

Case LXIII.—Thomas Flanagen, June 6, 1815. Phymosis discharge, gonorrhœa, ardor urinæ. He stated that he had been three months disordered, and that he had taken eighty mercurial pills which affected his mouth severely. Solut. Antim. Lot. fl. 19th, The discharge from within the prepuce had ceased, the gonorrhœa continued. He was directed to use an injection composed of one grain of Muriate of Mercury to six ounces of lime water, and, as he had no other symptom except gonorrhœa, he was discharged the hospital.

Case LXIV.—John Gaffney, June 7, 1815. Phymosis, swelling and inflammation of the penis, discharge, small ulcerated bubo in the right groin, considerable symptomatic fever. He stated that he had been eleven weeks disordered, and that he had used mercury, which slightly affected his gums.

gums. Venæsect. ad ℥ xvi. Solut. Antim. Fotus. Catapl. 13th, inflammation reduced. Lot. fl. 19th, discharged well.

Case LXV.—John Wheler, June 7, 1815. Several ulcers situated on the glans, corona, and internal prepuce, so extensive as to engage the greater surface of these parts, attended with a thickening of the prepuce, and a partial phymosis. He stated that he had been three months disordered, and that he used mercurial frictions until his mouth was severely affected, under which the ulcers healed, but were soon after succeeded by another crop.—Solut. Antim. Fotus. Catap. 13th, inflammation and disposition to phymosis subdued. Lot. fl. July 3d, the ulcers were healed; discharged the hospital.

Case LXVI.—Patrick Carroll, June 16, 1815. Excoriation of the glans and prepuce, discharge, gonorrhœa, swelling of the testis. He stated that he had been twelve months disordered, the commencement of which was gonorrhœa and ulcers on the glans, the cicatrices of which were apparent and extensive. Solut. Antim., Lot. flav. Fotus. 26th, excoriation healed; the swelling of the testis reduced. July 11th, discharged well.

Case LXVII.—John Williams, June 16, 1815. Permanent phymosis without inflammation or discharge; papular eruption extending over the entire body, and approaching nearly to the pustular form. He stated that he had been six months disordered, that the first appearances were ulcers on the penis which healed while under the influence of a severe course of mercury. Decoct. Sarsap. Solut. Antim. 26th, the eruption was declining, but he was affected with the chronic inflammation of the eyes which attends this eruption. July 17th, he was discharged well.

Case LXVIII.—Patrick Tracy, June 20, 1815. Phymosis, extreme inflammation and swelling of the penis, profuse discharge; the prepuce was of that livid colour which precedes mortification. He stated that he had been a month disordered, the first appearance was an ulcer on the frenum and prepuce; that he had used mercury extensively which affected his mouth; that the penis became painful, inflamed, and swelled three days previous to his admission; and that, on the morning he applied at the hospital, there was a profuse bleeding from the penis, which relieved him in a great degree from the severe pain he was suffering. Solut. Antim. Fotus. Catapl. 26th, more than one half of the prepuce was in a state of slough; he had daily profuse hæmorrhage from the ulcer, but as the swelling of the penis and symptomatic fever still continued I did not endeavour to check it. July 3d, the greater part of the prepuce and a large portion

 of of the glans had sloughed away; the remainder of the prepuce formed a useless mass at the frenal side of the glans. The pain and swelling were removed, and the sore which was now exposed exhibited a granulating surface. From this period, the ulcer gradually improved, and was healed before the 7th of August; and on the 14th, he was discharged.

Case LXIX.—Edward Mergin, June 24, 1815. Inflammation of the fauces, enlargement of the tonsils, which exhibited a clean red surface, condylomatous excrescences about the anus. He complained of pains in his knees and elbows, and stated that he had been affected during the preceding November with ulcers on the penis, which healed under a severe mercurial course; that two months afterwards his throat became affected and the pains followed. Decoct. Sarsap., Solut. Antim., Lot. fl. July 11th, the soft flat tumours in the neighbourhood of the anus had disappeared under the use of the yellow wash and the medicines directed; his throat was also improved, but he complained that the severity of the pains was unabated; the pills of Antimonial powder and Calomel so often mentioned were directed. July 23d, discharged well.

Case LXX.—Andrew Theogh, June 28, 1815. Phymosis, discharge, large bubo in the left groin containing matter. He stated that he had been seven weeks disordered, that the first symptoms was gonorrhœa, which left him afterwards without the aid of medicine or injection, and that he had not used mercury. Solut. Antim., Lot. fl. The bubo was freely opened. July 10th, the discharge had ceased. 23d, the bubo nearly healed; discharged the hospital.

I have now detailed the several cases of that class of venereal diseases which produce the papular eruption as their constitutional symptom; and which were under my care in the Lock and Richmond Hospitals, from the publication of my Essay till the end of last June. The reader may judge whether the diagnosis is not sufficiently clear and manifest; a difficulty, however, sometimes occurs which it is necessary to point out. If the ulcers happen to be concealed by phymosis, it is not easy, and indeed may sometimes be impossible, to ascertain their character until this symptom is removed; but the quality of the discharge, and the presence of induration may, in some degree, serve as guides. If the former be thin and ichorous, attended with considerable pain, we may presume that the concealed ulcer is phagedenic, but the phagedenic ulcer is in general so extensive, that it is easily distinguishable. If induration be perceptible beneath the prepuce, it may be inferred that the ulcer is chancrous; yet, yet, even on this point, we may be deceived, as the induration may be caused by warts. True chancre is, however, of so indolent a nature that it seldom excites inflammation or phymosis. If there be no induration, nor the discharge such as I have described, but purulent, we may conclude that the phymosis is occasioned by the virus, which has been the subject of this paper; and the instances I have detailed may lead us to the presumption, that, in the great majority of cases, phymosis is a symptom of this class of venereal diseases; indeed, so marked are the symptoms arising from this very prevalent poison, that in the vast number of cases which I have noted as appertaining to this class, I have witnessed but one which I ought to have arranged under another head, and this was a case of chancre concealed by a phymosis. The antimonial solution and yellow wash were employed; and, after the lapse of a fortnight, the patient was enabled to retract the prepuce, which exposed the callous cicatrix of a chancre; and, in the course of ten days afterwards he became affected with that slow, but well-marked, feverishness, which is so constantly attendant upon constitutional syphilis. Nocturnal pains and a node situated upon the clavicle succeeded. He was put upon a course of mercury, and these complaints were rapidly removed: and here let me again repeat, that mercurial courses ought never to be recurred to, until the disease has been ascertained, as in the present instance, to be syphilitic.

Notwithstanding my endeavours to compress the above detail of cases, they have swelled this paper to an unconscionable length; but the other species of venereal complaints are so much less frequent than that which has occupied us so long, that I hope the cases which I have to relate, respecting their symptoms and mode of cure, may be brought into a much narrower compass. I shall forward them to you in time for your next publication.

*Dublin; Aug.* 20, 1815. RICHARD CARMICHAEL.

The great mass of evidence contained in seventy well authenticated cases render unnecessary any apology for the length of Mr. Carmichael's paper, and we must impress our readers with the same sense of gratitude to the author as we have felt. Henceforth we hope to hear no more of the impossibility of finding discriminating characters in cases where the question is no less than the exhibition of a remedy which confounds all character, and has proved destructive in many complaints which would have healed spontaneously or yielded to mild remedies.* We hope also those much

* "Decessio ejus sua sponte; symptomata frequenter graviora, hydrargyro etiam caute adhibito."—*Dr. Burder's Remarks on Mr. Ferguson's Paper in the 4th Volume of Medico-Chirurg. Transactions.*

too general terms of *pseudo-syphilis and syphiloides*, which remind us of the early and more imperfect state of botany, will gradually fall into disuse, and evince an improvement in medicine by giving way to descriptive names. On this, as on many other occasions, we might recommend that some attention should be paid to what is called the vulgar or plebeian language. The privates, it seems, denominated the Lusitanean disease "the black pox," from a conviction on their own senses that it was different from those chancres which they had been accustomed to see. If the learned object to acquiring knowledge from such sources, let them examine Celsus, whom Hunter has almost translated without any knowledge of the original—so simple are the phenomena of nature, when described by adepts. We wish those writers who favour us with Arabic, Moorish, and Greek names for complaints which we can associate with nothing that now appears, would follow the arrangement of that esteemed ancient, in his chapter *de obscœnarum partium vitiis*, which is not only intelligible but applicable to all the local complaints on those parts described by modern authors, with the exception only of the true chancre.—Edit.

---

*For the London Medical and Physical Journal.*

